# Supplementary material for: Chromosome-level genome assembly and population genomic resource to accelerate orphan crop lablab breeding
Source: Nat Commun. 2023 Apr 17;14:1915. doi: 10.1038/s41467-023-37489-7 (PMC10110558; doi:10.1038/s41467-023-37489-7)
Supplement: Supplementary file 1 — Supplementary Information [file 41467_2023_37489_MOESM1_ESM.pdf]

Chromosome-level genome assembly and population genomic resource to accelerate orphan crop lablab breeding

Njaci *et al.*

Supplementary Note 1. Filtering for true-to-type genotypes in historic genebank collection

The lablab accessions used for evaluating global diversity in this study were acquired from different sources and conserved *ex situ* as seeds in the ILRI forage genebank, the earliest since 1982, with periodic monitoring for viability and regeneration for renewal of the seeds. These periodic genebank management practices involve risks to the genetic integrity of the accessions through pollen contamination, seed contamination, segregation, mislabeling, and other factors (e.g. as described in Chebotar *et al*.[1]). Hence, it was necessary to ensure the genetic integrity of plants within accessions and avoid potential contaminants before the genetic diversity analysis. Using pairwise IBD (Identity-By-Descent) analysis, plants within accessions were classified into “true-to-type”, “progeny”, or “contaminant” based on a PI_HAT[2] value of above 0.80, between 0.125 and 0.80, or less than 0.125, respectively. Six accessions with a single plant each were excluded from the analysis.

For eight accessions, all plants were unrelated to each other, and therefore considered “contaminants”. Out of the remaining 195 accessions, 124 were 100% true-to-type, indicating that there was no cross-pollination or seed mixing. Twenty-six accessions had a mixture of true-to-type and their progeny, indicating that some level of cross-pollination or segregation had taken place in this group. Another 25 accessions had a mixture of true-to-type and contaminants, and other 20 accessions had a mixture of the true-to-type, their progenies, and contaminants (Supplementary Figure 12). After removing contaminants and accessions whose clustering did not match previously obtained phenotype and/or molecular data, a total of 1552 plants from 191 accessions were retained. From the multiple plants per accession, one true-to-type individual plant with the least missing value was selected and used in genetic diversity and genome-wide association studies.

Supplementary Table 1. Summary of Nanopore reads statistics.

Sequencing metric	Value
Number of reads	4,678,100
Mean read length (bp)	6,071
Median read length (bp)	2,119
N50 (bp)	18,828
N75 (bp)	6,702
L50	386
L75	1,019,375
Longest read length (bp)	292,841
Shortest read length (bp)	76
No of runs	4

Supplementary Table 2. Comparison of assembly statistics for the lablab genome based on short reads and long reads.

Assembly metric	Short read assembly[3]	Long read assembly (this study)
Number of contigs/scaffolds	118,976	11
Total assembly length (bp)	395,472,305	417,870,439
N50 (bp)	621,673	38,128,793
L50	138	5
N75 (bp)	125,391	32,207,972
L75	491	8
Longest contig/scaffold (bp)	5,699,750	63,374,807

Supplementary Table 3. Summary statistics of genes identified in the lablab genome.

Annotation metric	All genes	Non-TE protein-coding genes
Number of genes	30,992	24,972
Number of transcripts	79,512	67,918
Number of CDS	79,512	67,918
Number of exons	473,986	450,920
Number of five prime UTRs	43,406	37,089
Number of three prime UTRs	45,371	36,674
Number of single exon Transcripts	14,225	8,132
Mean transcripts per gene	2.57	2.72
Mean exons per transcript	5.96	6.64
Mean gene length (bp)	4,136	4,627
Mean transcript length (bp)	4,175	4,574
Mean CDS length (bp)	1198.41	1317.7
Mean exon length (bp)	273.48	256

Supplementary Table 4. The number of TEs, TE families and the proportion of occupied assembly length by different classes of repeats identified and annotated in the lablab genome.

Class	Order	Superfamily	Number of TEs	Number of Families	Total %
Class I	LTR-RT	Copia	48,310	754	13.22
		Gypsy	19,746	185	4.72
		unknown	17,093	128	1.97
	LINE	unknown	396	5	0.03
Class II	TIR	CACTA	21,891	555	2.86
		hAT	15,632	264	1.65
		MUDR-Mutator	16,599	245	1.33
		PIF-Harbinger	814	23	0.06
		Tc1-Mariner	3,083	22	0.26
	MITE	CACTA	85	14	0.00
		hAT	878	49	0.06
		MUDR-Mutator	7,276	50	0.47
		PIF-Harbinger	286	6	0.01
		Tc1-Mariner	440	10	0.01
	Helitron	Helitron	15,645	43	1.47
Other	Unclassified repeat		100,741	927	15.24
Total			268,915	3,280	43.36

Supplementary Table 5. Types, amount and proportion of tandem repeats in the lablab genome.

Tandem repeats	Numbers	% of assembly
Microsatellite (2- 9 bp)	25,114	0.7
Minisatellites (10 -99 bp)	109,555	3.1
Satellites (>= 100 bp)	7,633	7.4
Total	142,302	11.2

Supplementary Table 6. Pairwise fixation index (Fst) among the seven major clusters (C) detected by the STRUCTURE analysis.

Clusters	Cluster I	Cluster II	Cluster III	Cluster IV	Cluster V	Cluster VI	Cluster VII
**Cluster I**							
**Cluster II**	0.97						
**Cluster III**	0.89	0.67					
**Cluster IV**	0.89	0.68	0.31				
**Cluster V**	0.73	0.94	0.85	0.86			
**Cluster VI**	0.88	0.87	0.46	0.39	0.92		
**Cluster VII**	0.88	0.88	0.52	0.54	0.93	0.76	

Supplementary Table 7. AMOVA showing the genetic variance among and within clusters.

Source of variation	Degrees of freedom (df)	Sum of squares	Mean sum of squares	Percentage of variation	*P*-value
Among clusters	6	277501.84	46250.306	81.04	0.001
Within clusters	155	76635.88	494.425	18.96	0.001

Supplementary Table 8. Minimum, maximum and average genetic divergence (Nei's D) between accessions within the seven clusters identified by STRUCTURE.

Clusters	Min.	Accessions	Max.	Accessions	Average
I	0.00437	ILRI_21083-M and ILRI_24796-M	0.40371	ILRI_13704-7 and ILRI_24800-M	0.185811
II	0.00035	ILRI_14430-2 and ILRI_14448-3	0.01808	ILRI_6930-10 and ILRI_14422-4	0.001903
III	0.00171	ILRI_13689-6 and ILRI_13699-2	0.06520	ILRI_18626-2 and ILRI_21049-M	0.042810
IV	0.00061	ILRI_14417-2 and ILRI_14456-3	0.06182	ILRI_21068-2 and ILRI_14483-3	0.035631
V	0.0135	ILRI_21081-3 and ILRI_24778-4	0.07977	ILRI_21048-5 and ILRI_24750.M	0.036293
VI	0.00058	ILRI_14435-3 and ILRI_14476-4	0.04062	ILRI_18593-7 and ILRI_14463-2	0.013194
VII	0.00052	ILRI_7379-6 and ILRI_14468-4	0.02218	ILRI_18603-5 and ILRI_18627-5	0.008637

Supplementary Table 9. Results of the analysis of variance (one-tailed) test for 14 quantitative traits among the six genetic clusters.

Trait	Abbreviation	N	Df	Sum_sq	Mean_sq	*F*-value	*P*-value	Unit	Further explanations
Time to flowering (50%)	d50fl	125	5	32.65	6.53	9.31	1.66E-07	days	At 50% of flowering
Flowering nodes	flownd	124	5	334.14	66.83	18.85	9.71E-14	number	Flowering nodes counted per inflorescence; number of nodes on the rachis
Flowering node density	flowndrat	92	5	48.59	9.72	20.38	2.30E-13	number/cm rachis	Density of flowering nodes on the rachis (no. nodes/rachis length)
Leaf length	leaflgth	125	5	46.40	9.28	4.65	6.50E-04	cm	Length of terminal leaflet at 50% flowering
Leaf ratio	leafrati	125	5	34.10	6.82	10.15	4.09E-08	ratio leaflgth/leafwdth	Leaflet length/leaf width
Leaf width	leafwdth	125	5	74.38	14.88	8.91	3.27E-07	cm	Width of terminal leaflet at 50% flowering
Peduncle length	peduncle	124	5	8002.74	1600.55	20.69	8.62E-15	cm	Length from branch to the first (bottom) flowering node
Plant height	plantht	118	5	35.31	7.06	13.23	3.98E-10	cm	At 100% of flowering
Pod length	podlgth	125	5	55.52	11.10	21.14	4.43E-15	cm	Length of fully developed pod prior to maturity
Pod ratio	podrati	125	5	66.86	13.37	32.77	6.81E-21	ratio podlgth/podwdth	Pod length/pod width
Pod width	podwdth	125	5	20.49	4.10	5.40	1.65E-04	cm	Width of fully developed pod prior to maturity
Average number of seeds per pod	seed_pod	125	5	62.08	12.42	36.14	2.29E-22	number	Seeds counted per fully developed pod prior to maturity
Seed mass (1000 seeds)	seedwt	125	5	34.09	6.82	10.27	3.34E-08	g/1000 seeds	Weight of g/1000 seeds
Seed yield	seedyld	88	5	32.43	6.49	9.80	2.28E-07	g/plant	At maturity

Supplementary Table 10. Results of the χ2 (one-tailed) test for seven qualitative traits among the six genetic clusters.

Trait	Abbreviation	N	χ2	Df	*P*-value	Unit	Further explanations
Flower colour	flowcol	125	58.286	5	0.00050	rating: 1 = white, 2 = coloured	At 50% of flowering
Plant growth habit	habit	92	72.951	5	0.00050	rating: 1 = erect, 2 = decumbent/ semi-erect, 3 = prostrate	At 2 months after establishment
Leafiness	leafines	92	23.396	5	0.28240	rating: 1 = low to 5 = high	Rating of leaf to stem ratio
Plant width	plantwth	92	25.700	5	0.01149	rating: 1 = narrow to 5 = wide	At 100% of flowering
Seed colour	seedcol	125	150.04	5	0.00050	rating: 1 = white/cream; 2 = grey; 3 = tan; 4 = redbrown; 5 = dark-brown; 6 = black	
Seed mottling	seedmott	92	36.595	5	0.00050	rating: 1 = no; 2 = yes	
Stem colour	stemcol	125	21.495	5	0.00150	rating: 1 = green; 2 = coloured	At 50% of flowering

Supplementary Table 11. Markers Trait Association summary from GWAS analysis.

Trait	Marker	Type	Chr	Position	MAF	FDR_adjusted P-value					R^2	effect
						GLM	MLM	MLMM	FarmCPU	BLINK		
Leaf length	42196183|F|0-35:A>G-35:A>G	SNP	Lp08	24038488	0.10	3.41E-03	-	1.30E-02	5.87E-03	5.12E-05	0.18	1.33
	42188186	SilicoDArT	Lp10	4707747	0.29	3.41E-03	-	-	2.31E-02	4.58E-03	0.19	-0.88
Leaf width	42196183|F|0-35:A>G-35:A>G	SNP	Lp08	24038488	0.10	1.31E-02	6.18E-02	3.02E-03	4.28E-03	6.31E-06	0.13	0.99
Leaf length-width ratio	42205202|F|0-43:C>T-43:C>T	SNP	Lp02	7611526	0.07	7.87E-04	-	-	6.48E-04	-	0.20	-0.90
	42196183|F|0-35:A>G-35:A>G	SNP	Lp08	24038488	0.10	4.19E-04	-	-	6.48E-04	-	0.18	0.87
Plant height	42187666	SilicoDArT	Lp03	47543687	0.40	5.30E-04	-	5.80E-04	1.50E-02	-	0.17	-0.46
	42201040	SilicoDArT	Lp04	30542557	0.23	4.19E-03	-	3.07E-02	5.67E-03	1.88E-03	0.10	-0.40
Days to 50% flowering	70185130	SilicoDArT	Lp03	48868491	0.39	1.46E-04	-	-	-	7.43E-04	0.24	-0.55
	42199202|F|0-56:G>C-56:G>C	SNP	Lp03	9993233	0.13	1.46E-04	-	-	-	4.61E-03	0.22	0.72
Pod length	42200209|F|0-23:G>T-23:G>T	SNP	Lp06	12569256	0.39	1.10E-02	-	-	-	6.92E-05	0.12	-0.57
Pod width	42194684	SilicoDArT	Lp04	6221408	0.46	4.61E-03	-	-	7.62E-03	-	0.17	-0.63
	42206028	SilicoDArT	Lp05	22796506	0.22	4.61E-03	-	-	1.40E-02	2.35E-03	0.17	0.60
	42213627	SilicoDArT	Lp06	26336574	0.30	7.38E-03	-	-	-	1.75E-05	0.15	-0.61
Pod length-width ratio	42195069	SilicoDArT	Lp03	3183668	0.30	8.40E-03	-	-	-	9.89E-05	0.11	-0.91
	42200824|F|0-21:T>C-21:T>C	SNP	Lp06	24304350	0.15	-	-	1.78E-03	4.40E-03	5.19E-08	0.10	0.45
	71243085	SilicoDArT	Lp06	2531646	0.12	-	-	-	7.13E-05	1.15E-06	0.07	0.33
	42207435	SilicoDArT	Lp07	5711942	0.22	2.68E-03	3.32E-02	6.97E-12	1.10E-06	2.49E-09	0.13	0.68
Thousand seeds weight	42205053|F|0-8:G>C-8:G>C	SNP	Lp07	35806317	0.08	4.21E-03	-	6.65E-04	-	1.64E-02	0.15	-0.75

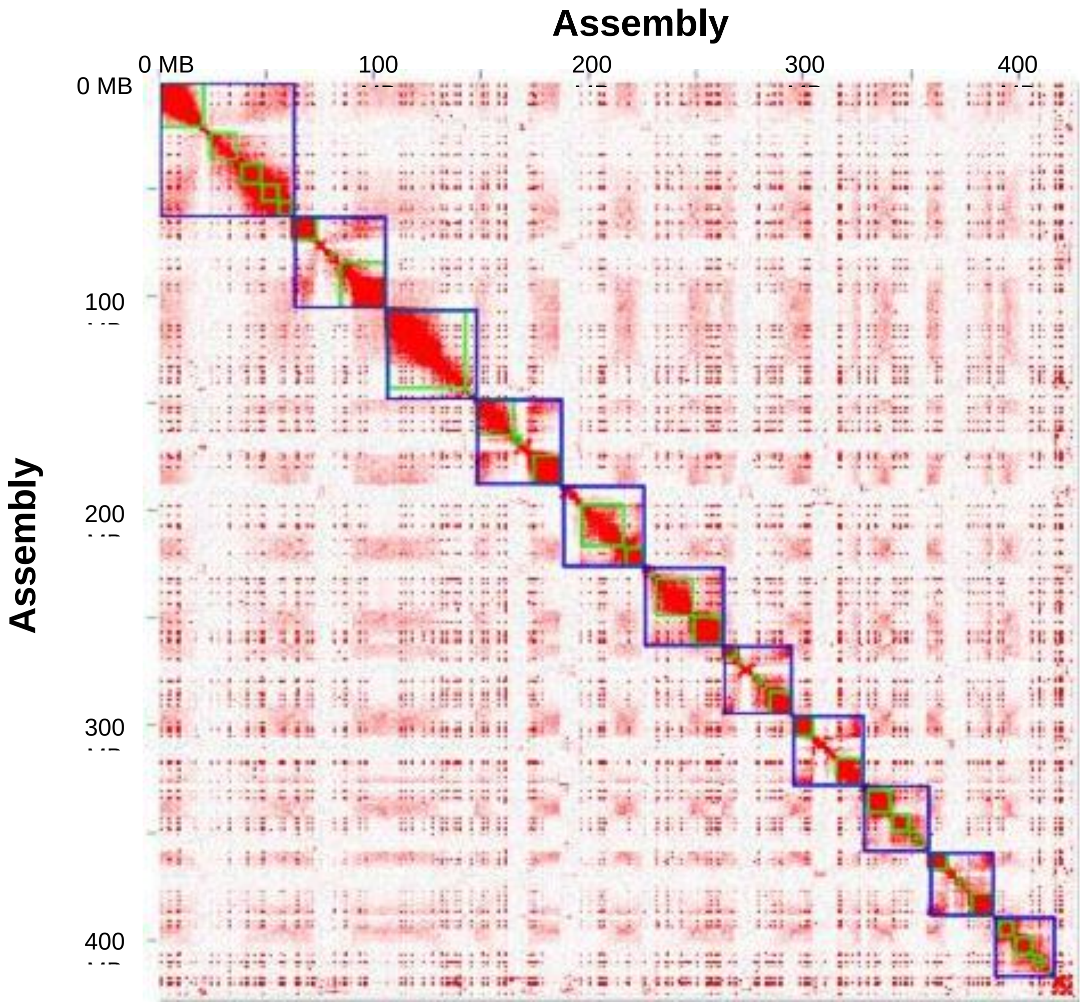

Supplementary Figure 1. HiC Scaffolding of the lablab genome. Hi-C contact map used for scaffolding the lablab assembly contigs to 11 chromosome. The blue boxes highlight the chromosomes while the green boxes highlight the contigs.

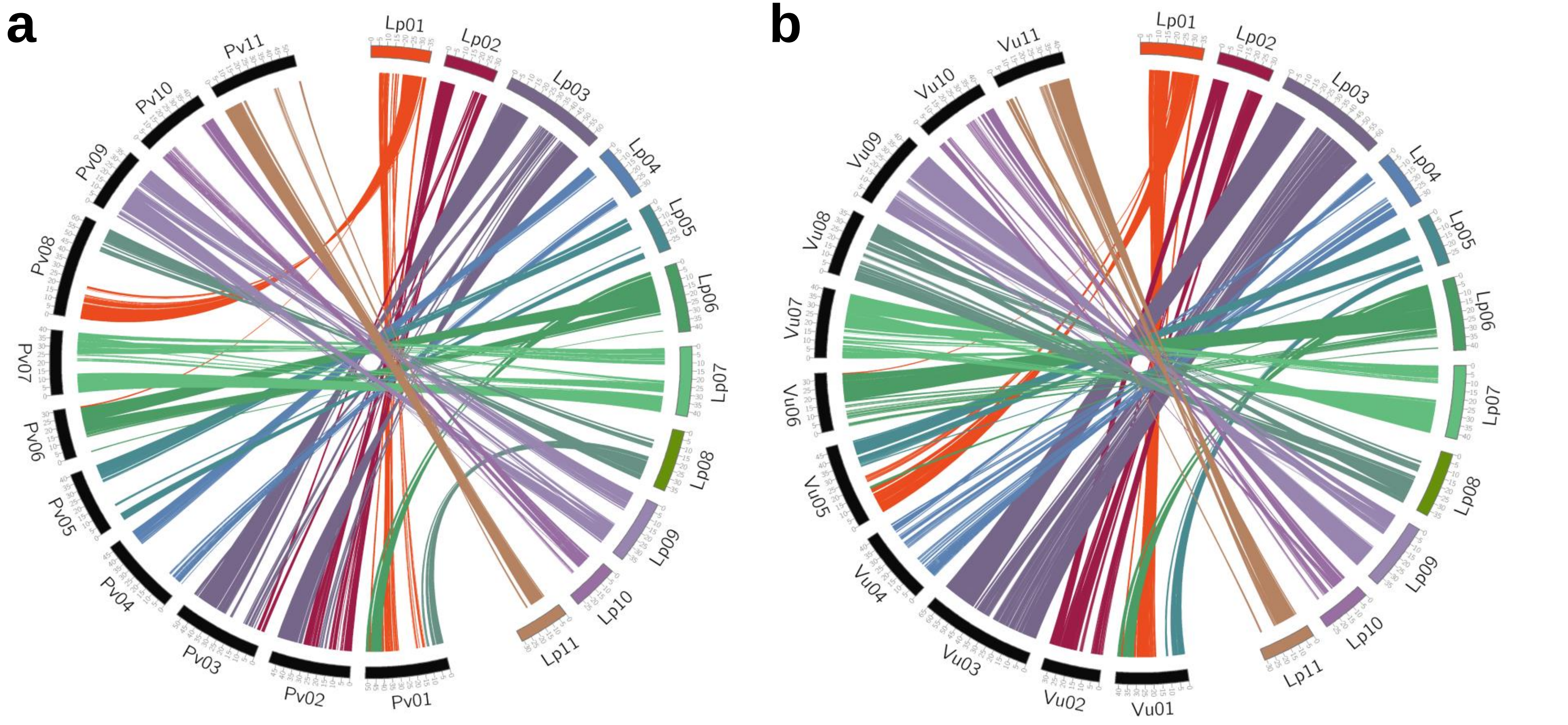

Supplementary Figure 2. Chromosome-level synteny of *Lablab purpureus* with related species. *L. purpureus* chromosomes have been named according to synteny with *P. vulgaris* **(a)** and *V. unguiculata* **(b)** chromosomes.

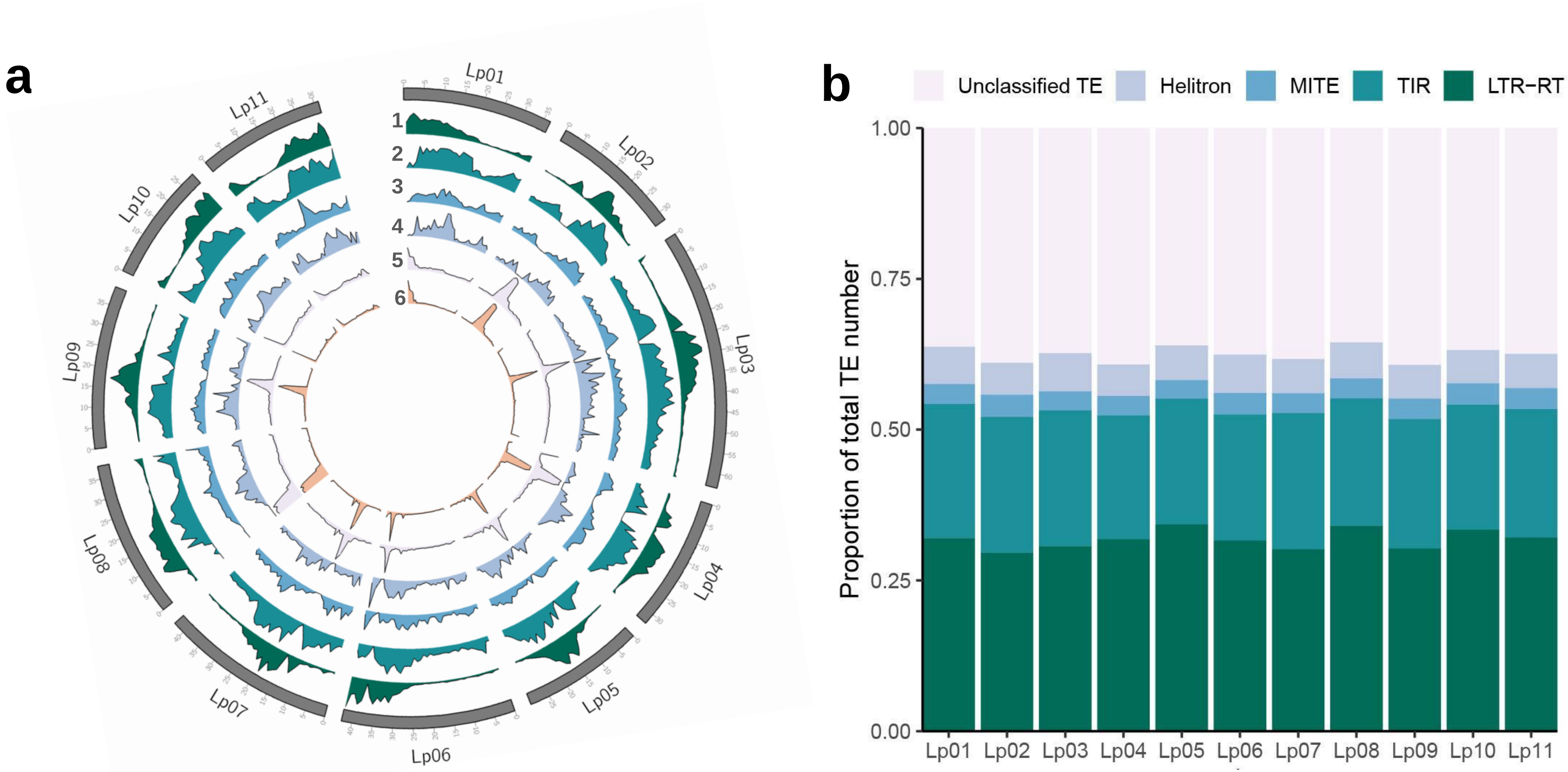

Supplementary Figure 3. Chromosomal repeat content in *Lablab purpureus*. (a) Relative densities of repeat elements along each chromosome. 1) Long Terminal Repeat RetroTransposons (LTR-RT), 2) Tandem Inverted Repeats (TIR), 3) Miniature Inverted Transposable Elements (MITE), 4) Helitron 5) Unclassified repeats, 6) Tandem repeats **(b)** Proportional abundance of identified transposable element orders on each chromosome. Source data are provided as a Source Data file.

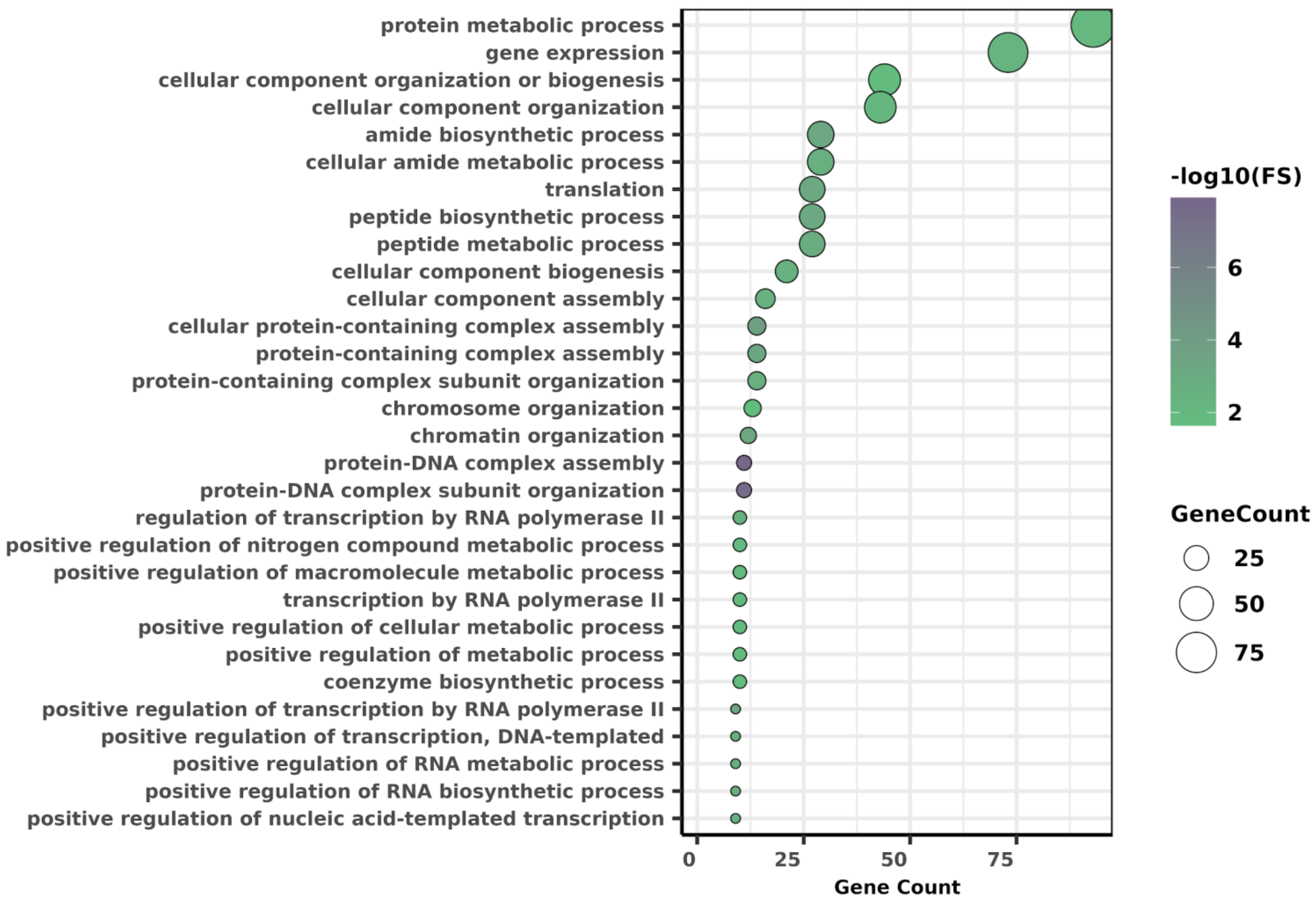

Supplementary Figure 4. Contracted gene families in lablab. Gene ontology terms enriched in the set of contracted gene families in lablab are indicted with size of the bubble proportional to the number of genes in that GO term. Source data are provided as a Source Data file.

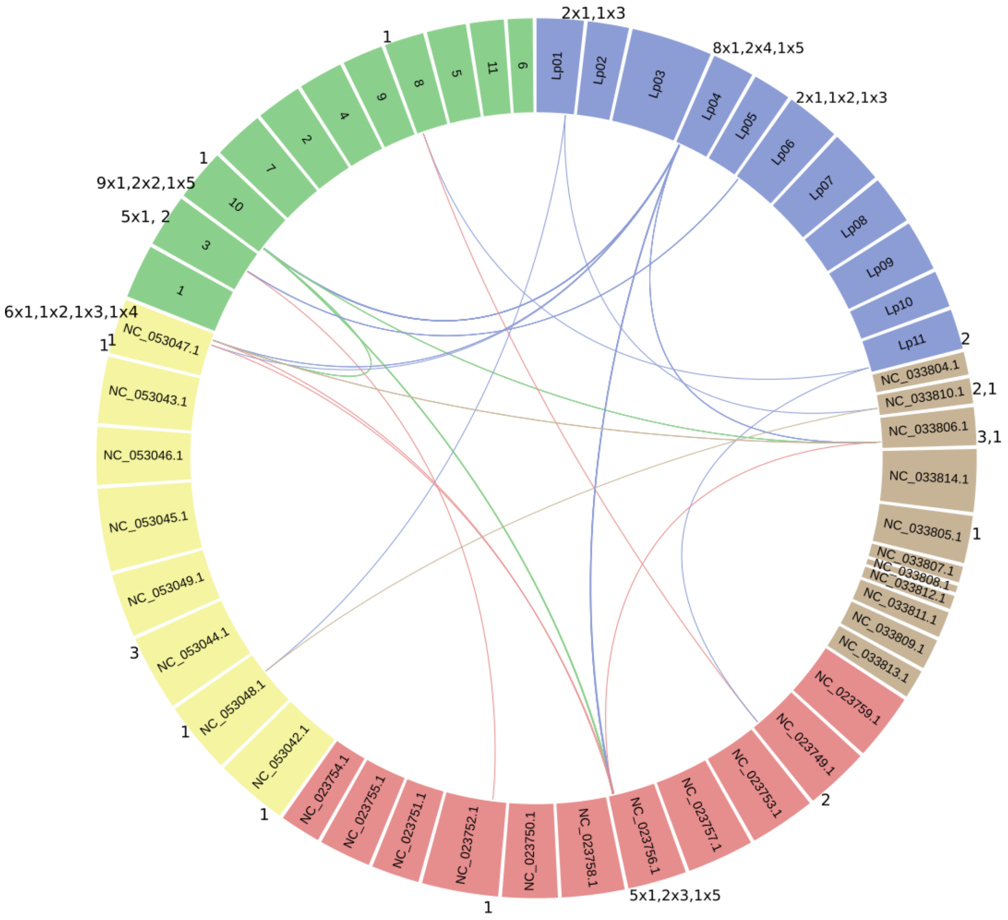

Supplementary Figure 5. Collinear relationship between trypsin inhibitors encoding genes in the genomes of lablab (purple), *Vigna angularis* (green) *Cajanus cajan* (brown), *Phaseolus vulgaris* (pink) and *Medicago truncatula* (yellow) are shown with connections in the middle. The number of trypsin inhibitor encoding genes or the length of the tandem array are indicated in the outer track.

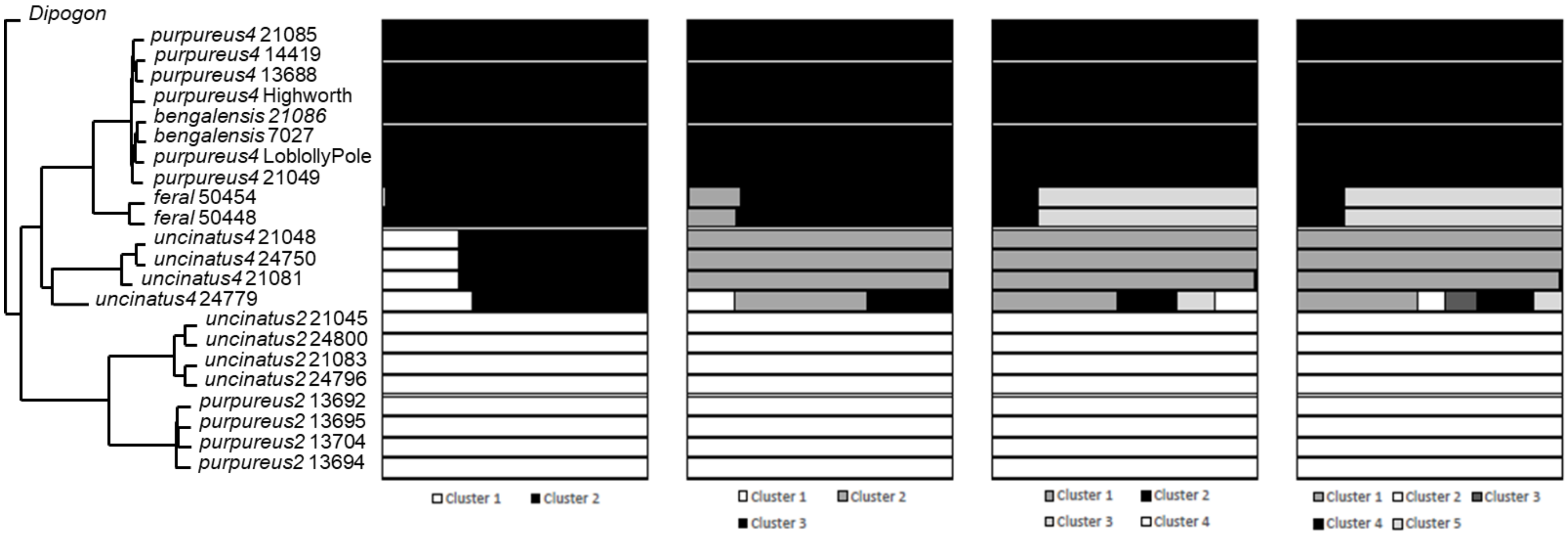

Supplementary Figure 6. STRUCTURE analysis varying the number of clusters from 2 to 5 (left to right). Individuals and relationships are shown on the left. Cluster membership is indicated according to the proportion of each bar in different shades. Source data are provided as a Source Data file.

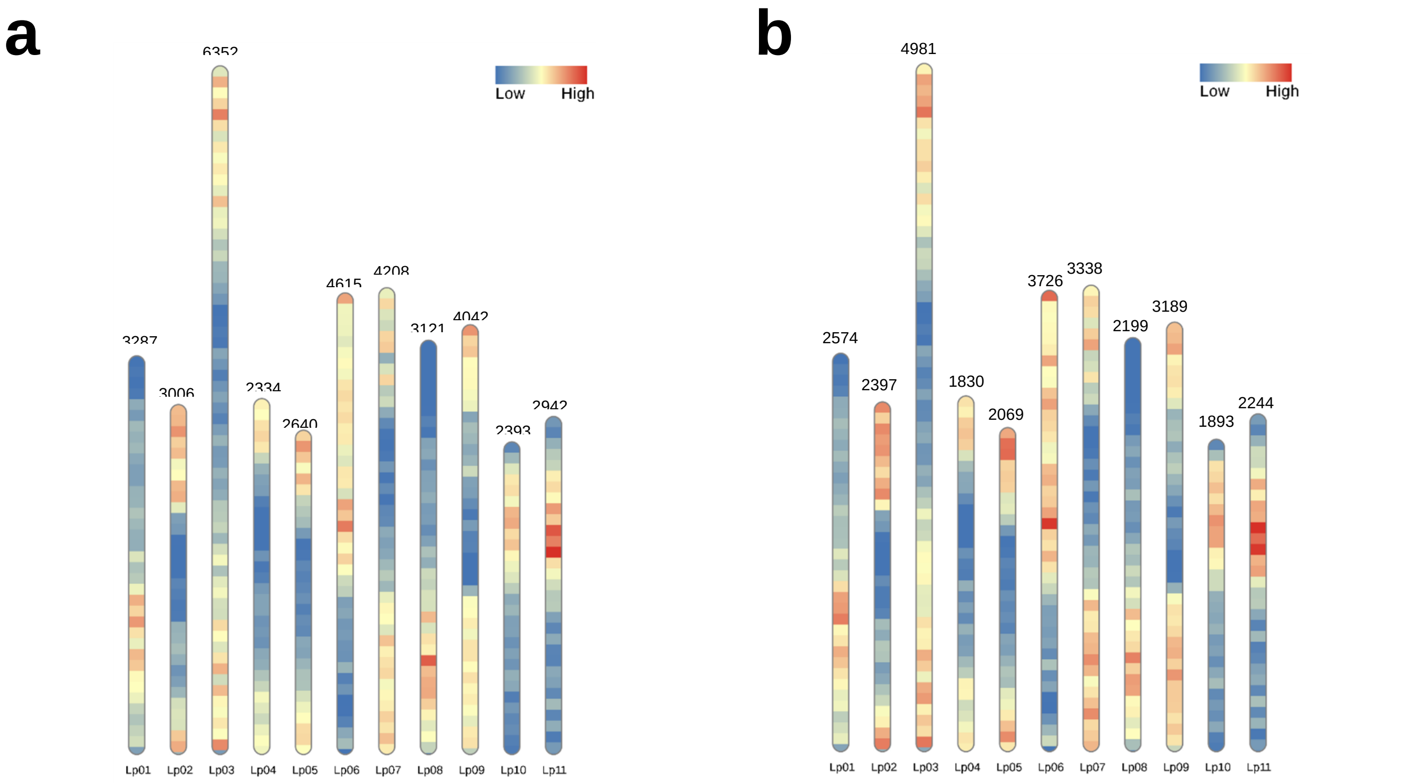

Supplementary Figure 7. GBS polymorphism in comprehensive lablab collection. Genome-wide distribution of SNPs (**a**) and SilicoDArTs (**b**) markers in 1 Mbp bins across the eleven chromosomes of the lablab reference genome. The total number of SNPs or SilicoDArT markers are presented above each chromosome. Source data are provided as a Source Data file.

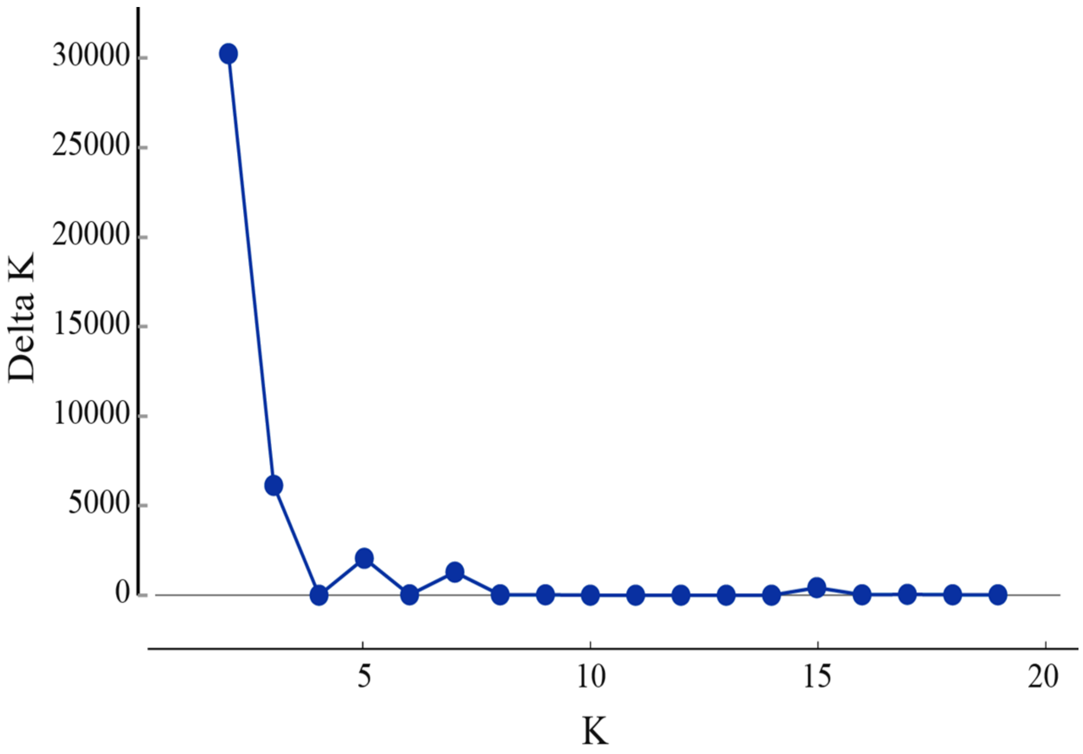

Supplementary Figure 8. The delta K suggesting two major groups and seven subgroups detected by the admixture model in STRUCTURE analysis. Source data are provided as a Source Data file.

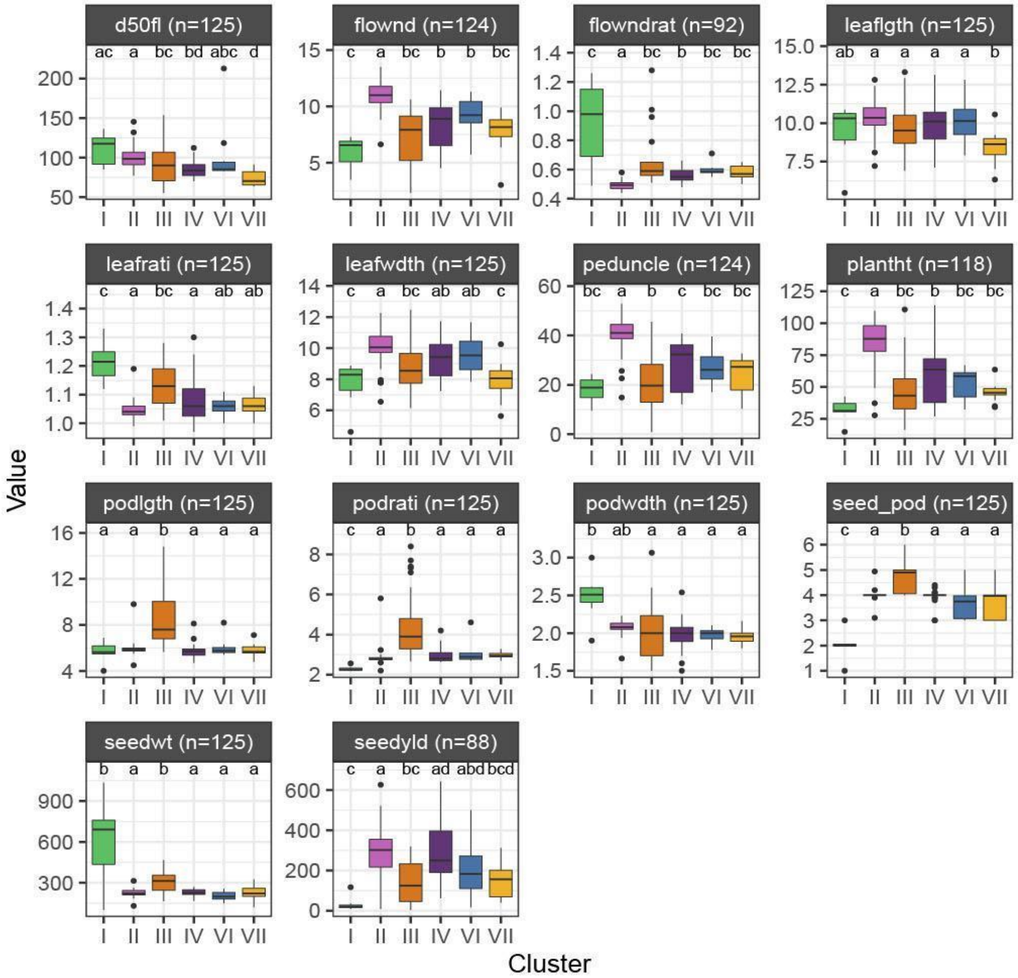

Supplementary Figure 9. Quantitative phenotypic variation in a comprehensive lablab collection. Boxplots showing phenotypic variation of different morpho-agronomic quantitative traits among the genetic clusters identified in lablab**.** The colours are according to the STRUCTURE analysis with k = 7, and trait abbreviations are explained in Table S14. The total number of accessions in each plot is indicated in parenthesis in the title. Colored boxes represent interquartile range (IQR) with the first and third quartiles at the lower and upper limits, respectively. the black mid-line in each box represent the median and the whisker represent 1.5*IQR. The letters above each box show the TUKEY multiple comparison groupings. Clusters with the same letter are statistically different trait distribution. Source data are provided as a Source Data file.

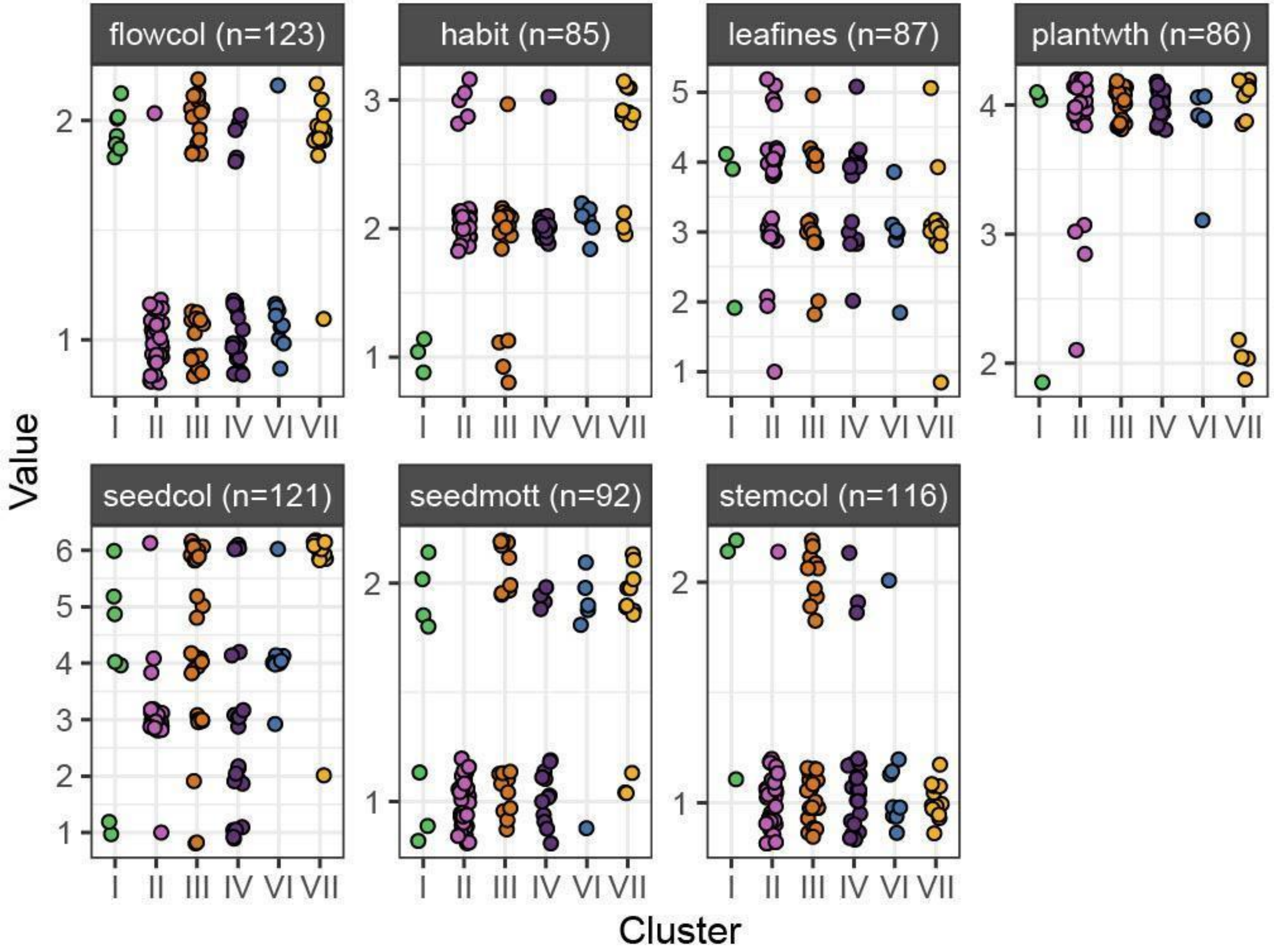

Supplementary Figure 10. Qualitative phenotypic variation in a comprehensive lablab collection. Jitter plots showing phenotypic variation of seven qualitative traits among the genetic clusters identified in lablab. The colors are according to the STRUCTURE analysis with k = 7, and trait abbreviations are explained in Table S15. The total number of accessions in each plot is indicated in parenthesis in the title. Identical categorical data points are randomly scattered on the x and y axis. Source data are provided as a Source Data file.

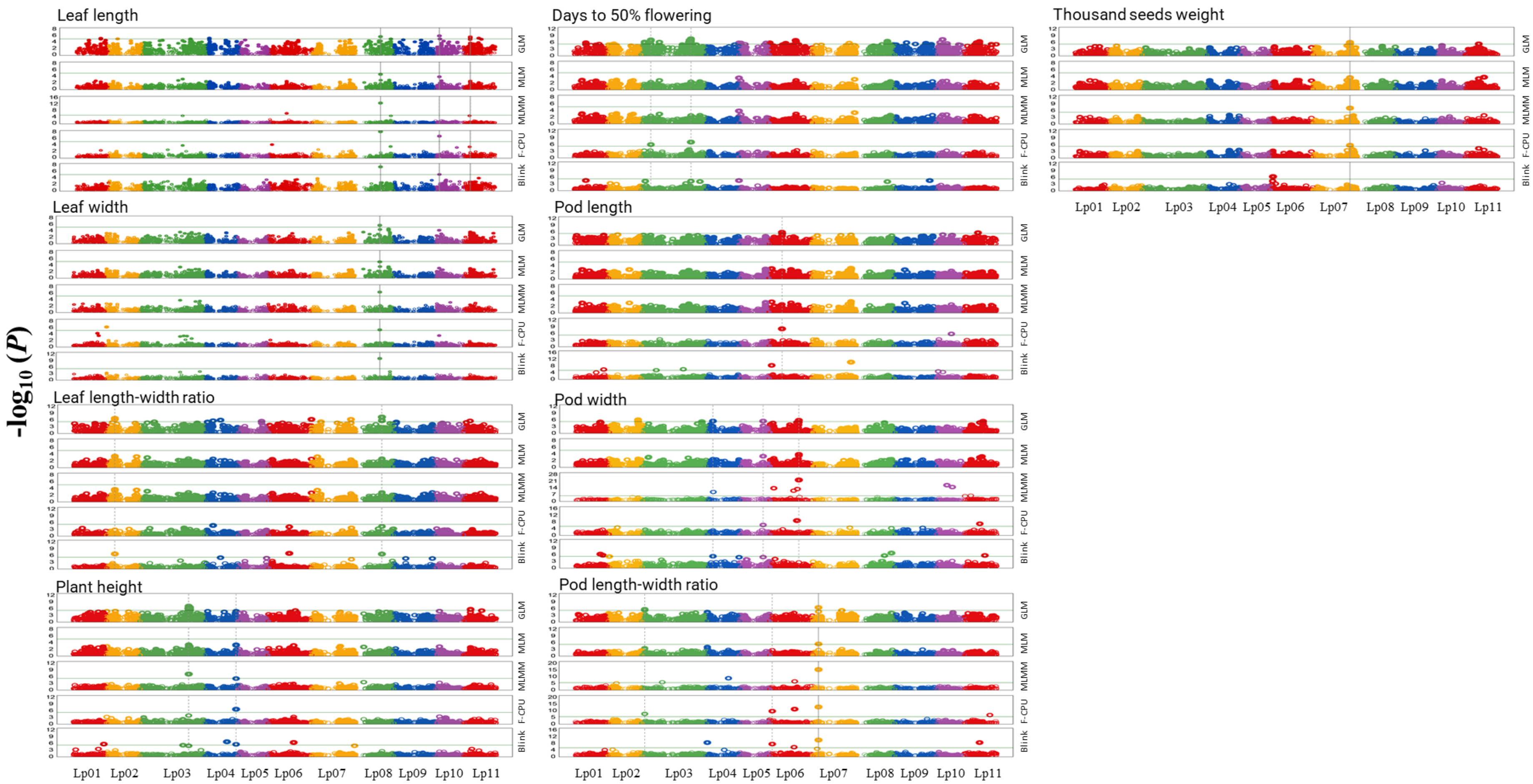

Supplementary Figure 11. Manhattan plots showing the distribution of significant marker-trait association (MTA) identified in the lablab genome for nine quantitative traits (Table S16). Vertical axis at the left represents the -log10 *p*-value, and markers and their chromosome positions are shown in horizontal axis. The five GAPIT models (Blink, FarmCPU, MLMM, MLM, GLM) used are shown at right vertical axis.

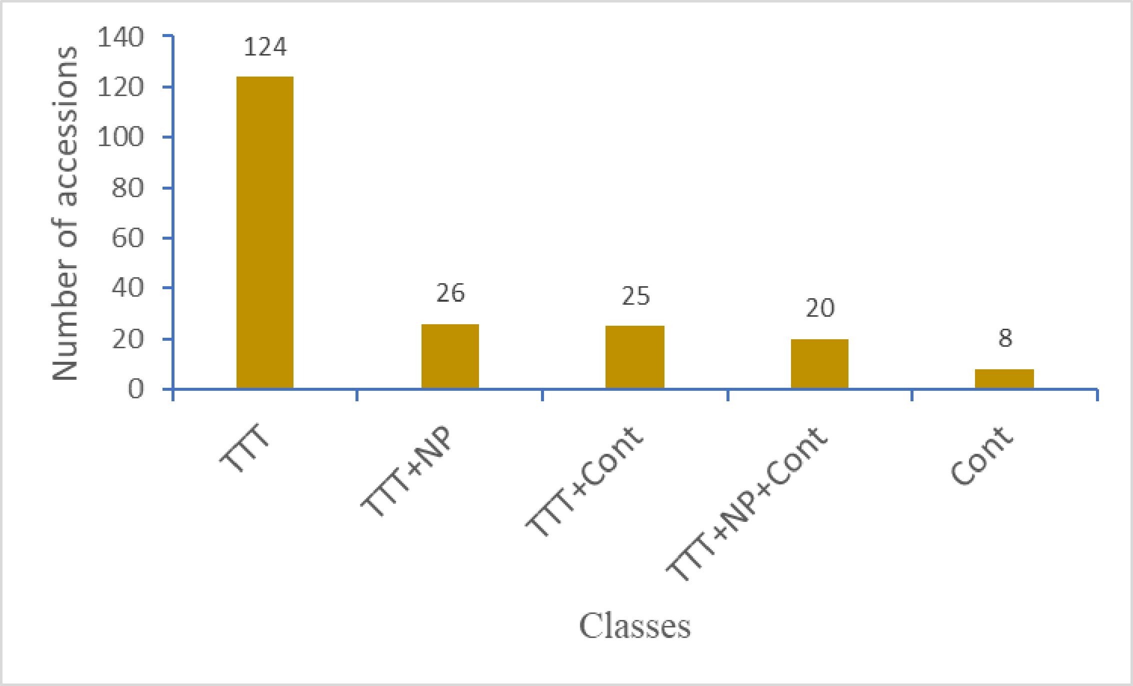

Supplementary Figure 12. Identity-By-Descent classification of global lablab collection. Number of accessions classified as true-to-type (TTT), true-to-type and progenies (TTT+NP), true-to-type and contaminants (TTT+Cont), true-to-type and progenies and contaminants (TTT+NP+Cont), and accessions with 100% contaminants (Cont)**,** based on a pairwise Identity-By-Descent (IBD) analysis.

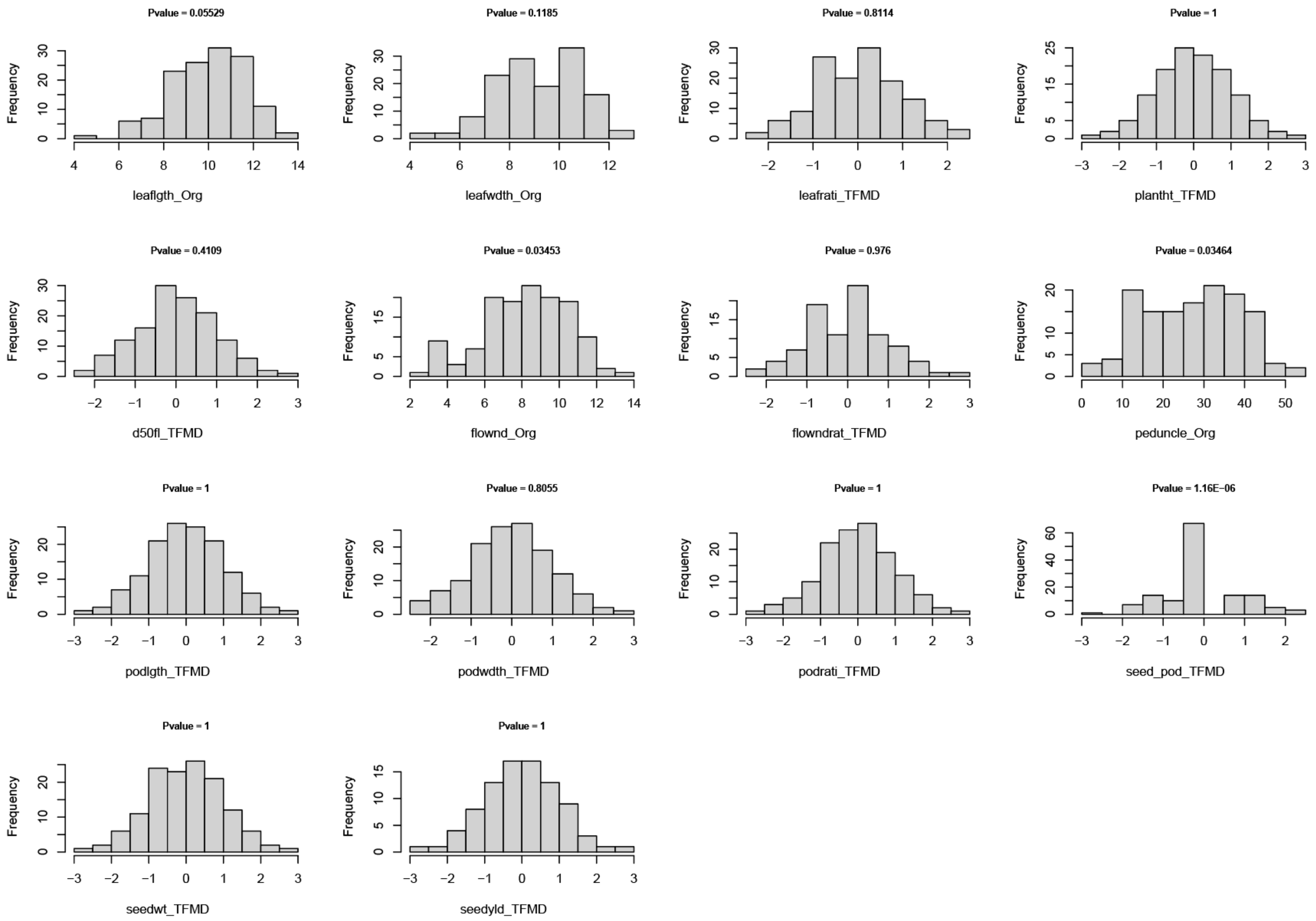

Supplementary Figure 13. Distribution of the historical phenotype data of quantitative traits. When a trait's distribution was closer to normal ($P > 0.01$), the original data (org) were used; however, the data were transformed (TFMD) whenever the distribution significantly ($P < 0.01$) deviated from normality. One-tailed Shapiro-Wilk test was used to test for normality.

Supplementary references

[1] Chebotar, S. *et al.* Molecular studies on genetic integrity of open-pollinating species rye (*Secale cereale* L.) after long-term genebank maintenance. *Theor Appl Genet* **107**, 1469-1476 (2003).

[2] Purcell, S. *et al.* PLINK: A tool set for whole-genome association and population-based linkage analyses. *Am J Hum Genet* **81**, 559–575 (2007).

[3] Chang, Y. *et al.* The draft genomes of five agriculturally important African orphan crops. *Gigascience* **8**, giy152 (2019).
