# Supplementary material for: Chromosome-level genome assembly and population genomic resource to accelerate orphan crop lablab breeding
Source: Nat Commun. 2023 Apr 17;14:1915. doi: 10.1038/s41467-023-37489-7 (PMC10110558; doi:10.1038/s41467-023-37489-7)
Supplement: Supplementary file 3 — Description of Additional Supplementary Files [file 41467_2023_37489_MOESM3_ESM.pdf]

Description of Additional Supplementary Files

File Name: Supplementary Data 1
Description: Orthogroups in lablab and other legumes

File Name: Supplementary Data 2
Description: GO annotation of lablab-specific gene clusters

File Name: Supplementary Data 3
Description: GO annotation of gene families expanded in lablab

File Name: Supplementary Data 4
Description: Details and sequencing statistics of resequencing samples

File Name: Supplementary Data 5
Description: Lablab accessions genotyped for genetic diversity study
