# Supplementary material for: Chromosome-level genome assembly and population genomic resource to accelerate orphan crop lablab breeding
Source: Nat Commun. 2023 Apr 17;14:1915. doi: 10.1038/s41467-023-37489-7 (PMC10110558; doi:10.1038/s41467-023-37489-7)
Supplement: Supplementary file 9 — Reporting Summary [file 41467_2023_37489_MOESM9_ESM.pdf]

# nature portfolio

Corresponding author(s): Oluwaseyi Shorinola, Mark Chapman and Chris Jones

Last updated by author(s): Feb 20, 2023



# Reporting Summary

Nature Portfolio wishes to improve the reproducibility of the work that we publish. This form provides structure for consistency and transparency in reporting. For further information on Nature Portfolio policies, see our Editorial Policies and the Editorial Policy Checklist.

## Statistics

For all statistical analyses, confirm that the following items are present in the figure legend, table legend, main text, or Methods section.

| n/a | Confirmed | |
|---|---|---|
| ☐ | ☒ | The exact sample size (*n*) for each experimental group/condition, given as a discrete number and unit of measurement |
| ☒ | ☐ | A statement on whether measurements were taken from distinct samples or whether the same sample was measured repeatedly |
| ☐ | ☒ | The statistical test(s) used AND whether they are one- or two-sided<br>*Only common tests should be described solely by name; describe more complex techniques in the Methods section.* |
| ☒ | ☐ | A description of all covariates tested |
| ☐ | ☒ | A description of any assumptions or corrections, such as tests of normality and adjustment for multiple comparisons |
| ☐ | ☒ | A full description of the statistical parameters including central tendency (e.g. means) or other basic estimates (e.g. regression coefficient) AND variation (e.g. standard deviation) or associated estimates of uncertainty (e.g. confidence intervals) |
| ☐ | ☒ | For null hypothesis testing, the test statistic (e.g. *F*, *t*, *r*) with confidence intervals, effect sizes, degrees of freedom and *P* value noted<br>*Give P values as exact values whenever suitable.* |
| ☒ | ☐ | For Bayesian analysis, information on the choice of priors and Markov chain Monte Carlo settings |
| ☒ | ☐ | For hierarchical and complex designs, identification of the appropriate level for tests and full reporting of outcomes |
| ☒ | ☐ | Estimates of effect sizes (e.g. Cohen's *d*, Pearson's *r*), indicating how they were calculated |

*Our web collection on statistics for biologists contains articles on many of the points above.*

## Software and code

Policy information about availability of computer code

| | |
|---|---|
| Data collection | Data collection did not involve the use of software or code. |
| Data analysis | Data analysis required Guppy basecaller (ver 4.1.1), Flye de novo long reads assembler (ver 2.7.1), HyPo hybrid polisher (ver 1), BUSCO (ver 4.0.6 and 5.2.2), Trinity (ver 2.8.5), Funannotate (ver 1.8.7), PASA (ver 2.4.1), Augustus (ver 3.3.3), SNAP (ver 2006-07-28), Glimmerhmm (ver 3.0.4), Evidence modeller (ver 1.1.1), tRNAscan-SE49 (ver 2.0.9), plant.annot (github.com/PGSB-HMGU/plant.annot; commit f515c11, June 2019), HISAT (ver 2.1.0), Stringtie (version 1.2.3), GenomeThreader (ver 1.7.1), Transdecoder53 (ver 3.0.0), hmmscan (ver 3.1b2), AHRD (ver 3.3.3), EDTA (ver 1.9.7), Tandem RepeatFinder (ver 4.09), Orthofinder (ver 2.4), CAFE (ver 5), BLAST (ver 2.2.30+), Trimmomatic (ver 0.32), Bowtie2 (ver 2.2.3), SAMtools (ver 1.1), MCScanX (Nov 1 2022 commit), MUSCLE (ver 3.8.1551), FastTree (ver 2.1.11), iTol (v 6.3), Kallisto (ver 0.44.0), DESeq2 (ver 1.24.0), Pheatmap (ver 1.0.12), Picard toolkit (ver 2.8.3), bcftools (ver1.6.0), vcftools (ver 0.1.14), VCF2Dis (github.com/BGI-shenzhen/VCF2Dis/; ver 1.36), STRUCTURE (ver 2.3.4), R (ver 4.1.2), QUAST (ver 5.0.2), GAPIT (ver 3), samblaster (ver 0.1.24), bwa-mem (ver 0.7.17-r1198-dirty), Juicebox (ver 1.11.08), fastp (ver 0.23.2) |

For manuscripts utilizing custom algorithms or software that are central to the research but not yet described in published literature, software must be made available to editors and reviewers. We strongly encourage code deposition in a community repository (e.g. GitHub). See the Nature Portfolio guidelines for submitting code & software for further information.

## Data

Policy information about availability of data

All manuscripts must include a data availability statement. This statement should provide the following information, where applicable:

- Accession codes, unique identifiers, or web links for publicly available datasets
- A description of any restrictions on data availability
- For clinical datasets or third party data, please ensure that the statement adheres to our policy

The lablab genome and annotation files are available from eiDAL105 (http://dx.doi.org/10.5447/ipk/2022/26) and at https://hpc.ilri.cgiar.org/~bngina/lablab_longread_sequencing_March_2022/. Nanopore long reads used for the reference assembly are available from NCBI SRA under BioProject PRJNA824307. Illumina reads for the resequencing samples are available from the NCBI SRA under project numbers PRJNA834808. The following publicly available databases were used: UniProt Magnoliophyta, reviewed/Swiss-Prot (https://www.uniprot.org/), The Arabidopsis Information Resource (https://www.arabidopsis.org/), TrEMBL (http://www.bioinfo.pte.hu/more/TrEMBL.htm; ver. 37.2). Genome sequences of related legumes were downloaded from EMSEMBL (https://plants.ensembl.org/index.html). Genome version numbers are given in the text.

## Human research participants

Policy information about studies involving human research participants and Sex and Gender in Research.

| | |
|---|---|
| Reporting on sex and gender | Not applicable |
| Population characteristics | Not applicable |
| Recruitment | Not applicable |
| Ethics oversight | Not applicable |

Note that full information on the approval of the study protocol must also be provided in the manuscript.

# Field-specific reporting

Please select the one below that is the best fit for your research. If you are not sure, read the appropriate sections before making your selection.

☐ Life sciences ☐ Behavioural & social sciences ☒ Ecological, evolutionary & environmental sciences

For a reference copy of the document with all sections, see nature.com/documents/nr-reporting-summary-flat.pdf

# Ecological, evolutionary & environmental sciences study design

All studies must disclose on these points even when the disclosure is negative.

| | |
|---|---|
| Study description | We sequenced, assembled, and annotated the lablab genome; we carried out population genetic and evolutionary analyses into the crop's origin and diversification, including relating this to phenotypic measures. |
| Research sample | A single lablab accession (cv. Highworth) was used for genome sequencing. Highworth was used as it is a commercial cultivar and was previously sequenced using a short-read sequencing approach.<br><br>Resequencing used diverse samples from Africa, all are available from seed banks. Samples were selected based on fitting the key groups (2-seeded and 4-seeded; wild and domesticated). We selected a range of samples from the four groups of interest (cultivated 4-seeded, cultivated 2-seeded, wild 4-seeded, wild 2-seeded) to examine differentiation between these groups, as well as individuals of subsp. bengalensis and two likely feral samples where the taxonomic relationships were previously unclear. Short-read sequenciing files from cv Highworth were taken from the NCBI SRA.<br><br>Population Genetics - We analysed the majority of the lablab accessions that were collected from 25 countries in Africa, Asia, America, Australia, and Europe and maintained in the ILRI forage genebank in order to capture as much of the available genetic diversity of lablab. The population reflects most of the global diversity, including accessions from Africa, where the species originated and diversified. Hence, the four-seeded and two-seeded cultivated as well as wild accessions were included in the study.<br><br>Existing data from Pengelly, B. C. & Maass, B. L. (2001) Genet. Resour. Crop Evol. 48, 261–272 was also used. |
| Sampling strategy | The choice of sample for reference genome sequencing was based on previously determined reference cultivar (Chang et al, 2019, GigaScience) Samples sizes for the phylogenetics and population genetics were not predetermined and instead covered the key groups of interest, as follows:<br>Resequencing – we selected samples of diverse origin to reduce the chance of any findings being related to sampling closely-related germplasm.<br>Population genetics – From each of the four and two-seeded cultivated and wild lablab accessions, we genotyped as many as we |

| | |
|---|---|
| | could. The sample sizes are considered sufficient to cover the global genetic diversity as the accessions analysed originated from 25 different countries in Africa (the native range), Asia, America, Australia, and Europe. |
| Data collection | Pen and paper or electronic notebooks were kept to record methods by the researchers conducting each experiment (see author contributions section); all data was recorded electronically by the instruments and software named in the Data Collection section. |
| Timing and spatial scale | Newly generated data were from greenhouse grown plants and therefore time and scale is not relevant.<br>Phenotypic data collection is reported in the original articles by Pengelly and Maass (2001) and Wiedow (2001) |
| Data exclusions | No data were excluded from the analyses with the exception of (1) Dart-seq samples with high values of missing data using established cut-offs and (2) cultivars with contaminated seeds lot whose morphological and diversity data did not match previously described morphology and diversity data [Pengelly and Maass (2001) and Wiedow (2001)]. |
| Reproducibility | Bootstrap scores were used to examine support for the phylogenetic relationships. For GWAS, only associations supported by two or more association models are reported. Genetic and genomic data were generated and analysed once, with no need to replicate any sequencing or analyses. Seed are available from the genebanks listed in the article. |
| Randomization | Randomization is not relevant to this study. Samples are not pre-allocated to groups. Phenotypic data collection is reported in the original articles by Pengelly and Maass (2001) and Wiedow (2001) |
| Blinding | All genome sequencing data was generated from healthy plants from seed from seedbanks. No other selection or blinding was necessary or carried out. Sequencing data was collected electronically, with no potential for human bias.<br>Phenotypic data collection is reported in the original articles by Pengelly and Maass (2001) and Wiedow (2001) |

Did the study involve field work? ☐ Yes ☒ No

# Reporting for specific materials, systems and methods

We require information from authors about some types of materials, experimental systems and methods used in many studies. Here, indicate whether each material, system or method listed is relevant to your study. If you are not sure if a list item applies to your research, read the appropriate section before selecting a response.

## Materials & experimental systems

| n/a | Involved in the study |
|---|---|
| ☒ | ☐ Antibodies |
| ☒ | ☐ Eukaryotic cell lines |
| ☒ | ☐ Palaeontology and archaeology |
| ☒ | ☐ Animals and other organisms |
| ☒ | ☐ Clinical data |
| ☒ | ☐ Dual use research of concern |

## Methods

| n/a | Involved in the study |
|---|---|
| ☒ | ☐ ChIP-seq |
| ☒ | ☐ Flow cytometry |
| ☒ | ☐ MRI-based neuroimaging |
